# Current Animal Models of Cleft Lip and/or Palate: A Narrative Review

**DOI:** 10.3390/biomedicines14071437

**Published:** 2026-06-24

**Authors:** In-Won Chang, Shirley Zheng, Zhong Zheng, Anh D. Le, Chun-Hsi Chung, Myra F. Laird, Chenshuang Li

**Affiliations:** 1 Department of Orthodontics, School of Dental Medicine, University of Pennsylvania, Philadelphia, PA 19104, USA; inwonc@upenn.edu (I.-W.C.); chunc@upenn.edu (C.-H.C.); 2 School of Dental Medicine, University of Pennsylvania, Philadelphia, PA 19104, USA; shirley9@upenn.edu; 3 Department of Periodontics, School of Dental Medicine, University of Pennsylvania, Philadelphia, PA 19104, USA; leozz95@gmail.com; 4 Department of Oral and Maxillofacial Surgery/Pharmacology, University of Pennsylvania, Philadelphia, PA 19104, USA; anh.le@pennmedicine.upenn.edu; 5 Department of Basic and Translational Sciences, University of Pennsylvania, Philadelphia, PA 19104, USA; mflaird@upenn.edu

**Keywords:** cleft lip, cleft palate, animal models, craniofacial development, palatogenesis, teratogenic models, genetic models

## Abstract

Cleft lip with or without cleft palate (CL/P) is one of the most common congenital craniofacial anomalies worldwide and presents significant functional, esthetic, and psychosocial challenges. Despite advances in multidisciplinary care and surgical reconstruction, complications such as impaired wound healing, scar formation, and growth disturbances warrant the development of novel regenerative and surgical strategies, which heavily rely on animal models at the pre-clinical stage. For the current narrative review, the literature search was performed by combining cleft phenotype terms with modeling-approach terms in six databases and was supplemented by manual review of reference lists from full-text articles. The included articles were summarized based on cleft type and the methods for cleft induction (chemically induced, genetically engineered, and surgically created). Particularly, chemical teratogens such as retinoic acid, 2,3,7,8-tetrachlorodibenzo-p-dioxin (TCDD), corticosteroids, and 6-aminonicotinamide have been widely used to induce cleft phenotypes and elucidate environmental influences on palatogenesis, whereas genetic models have clarified the roles of key molecules and signaling pathways, including Sonic hedgehog (*SHH*), bone morphogenetic protein (*BMP*), and transforming growth factor-β (*TGF-β*), in the development of lip and palate. Meanwhile, the surgical models have focused on the alveolar cleft in skeletally mature animals for evaluating novel grafting materials. By comparing the strengths and limitations of existing models, this review highlights opportunities for improving experimental design and translational relevance in future cleft research. Overall, despite a wide range of CL/P animal models available, few replicate clinically relevant defect anatomy and the postnatal craniofacial deformation observed in CL/P patients, underscoring the need for the development of new models.

## 1. Introduction

Cleft lip with or without cleft palate (CL/P) is the second most common congenital craniofacial malformation, with an estimated prevalence of 1 in 600 births worldwide [1]. Beyond incidence, CL/P presents with a multi-tissue deficit involving mucosa, muscle, bone, and skin, which can result in feeding and breathing difficulties in newborns due to an impaired oral seal, ineffective swallowing, and nasal regurgitation. Functional sequelae also include hearing loss, speech problems, and substantial psychosocial influences [2,3,4,5].

Standard CL/P care is typically delivered through a staged surgical pathway with primary lip and palatal repair occurring when the patients are young [6,7,8]. Many patients also require additional procedures as they grow—such as lip revision, fistula management, palatal lengthening, pharyngeal flap/pharyngoplasty, columellar lengthening, rhinoplasty, septoplasty, and scar-related revisions—depending on functional deficits, esthetic concerns, and growth-related changes [6,9]. However, even when surgical timing and technique follow the currently accepted protocols, achieving durable healing and minimizing morbidity at cleft sites can be a challenge. The large tissue defect poses healing challenges in CL/P, associated with soft-tissue hypertrophic scarring and even wound dehiscence, with downstream consequences including infection risk and the need for additional costly operative intervention [10,11,12,13]. In addition, postoperative fibrosis may contribute to maxillary growth restriction and midfacial retrusion [14], and, consequently, a subset of patients ultimately require orthognathic correction such as Le Fort I osteotomy [15]. Hard-tissue reconstruction presents another set of limitations, including donor-site pain, delayed ambulation, and sensory complications [16], yet failure and the need for repeat grafting are still encountered [17,18]. When the alveolar segment remains insufficient, orthodontic tooth movement and subsequent prosthodontic rehabilitation can be compromised. Thus, despite multidisciplinary cleft teams and national initiatives aimed at improving outcomes, gains in clinical treatment outcome improvements over time have been uneven [19], reinforcing the need for strategies to improve tissue regeneration and reduce scar- and tension-driven complications, particularly around early revision procedures.

Progress in regenerative approaches depends on animal models that reproduce clinically relevant anatomy, timing, and biomechanical context while adhering to the 3R principles of animal research (Replacement, Reduction, and Refinement) [20,21]. Currently available review articles on animal models of cleft primarily focus on the genetic or epigenetic mechanisms underlying orofacial clefts [22,23,24,25,26], with limited attention to the practical aspects of model selection, establishment, and comparative evaluation across categories (chemically induced, genetically engineered, and surgically created). This review aims to summarize the available animal models for each type of CL/P and to explore gaps between these animal models and clinical scenarios.

## 2. Literature Searching and Selection

A literature search was conducted to identify animal models of cleft lip, alveolar cleft, and cleft palate reported in the peer-reviewed literature. Two categories of search terms were combined in each query, using a free-text Boolean (“Word 1” AND “Word 2”) strategy. The “Word 1” terms reflected the cleft phenotype of interest, and the “Word 2” terms reflected the modeling approach:

Word 1: “cleft lip and palate,” “cleft lip,” “cleft palate,” “alveolar cleft.”

Word 2: “animal model,” “small animal.”

**Figure 1:**
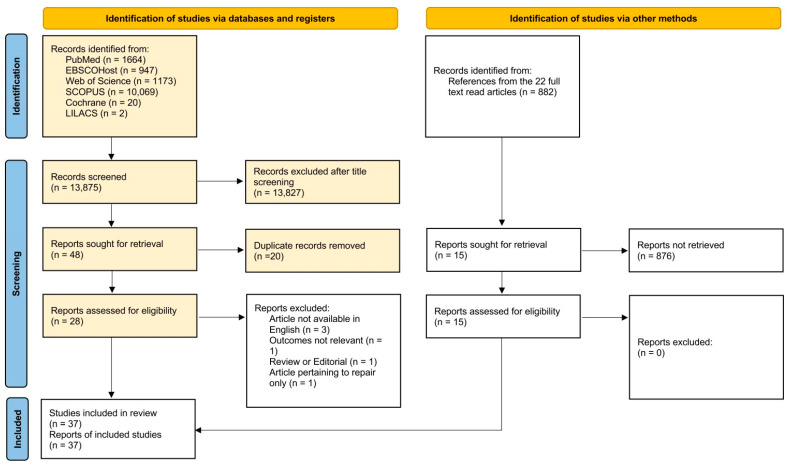
The PRISMA flow diagram demonstrating the study identification and screening processes.

Combining these terms produced eight keyword pairings that were searched across six databases—PubMed, EBSCOhost, Web of Science, Scopus, Cochrane, and LILACS. The initial database searching was completed in May 2025, which generated 13,875 records to be screened subsequently. The inclusion criteria comprised peer-reviewed studies, available in English, that described an animal model of cleft lip, cleft palate, cleft lip and palate, or alveolar cleft. Exclusion criteria were articles unavailable in English, outcomes not relevant to model development, review or editorial articles, conference abstracts, theses, and articles concerned solely with repair techniques. No limitation was set for the publishing date. Two investigators independently performed screening; in cases of discrepancies, a third investigator was consulted. The database search resulted in the inclusion of 22 articles. The literature search was further supplemented by a manual review of reference lists from the 22 full-text articles. After applying the same inclusive and exclusive criteria as listed above, 15 additional articles were included, resulting in 37 articles in total for further data extraction. The PRISMA flow diagram summarizing the screening process is presented in Figure 1.

Following article selection, data were extracted from each of the 37 included studies and tabulated by cleft phenotype as presented in Table 1, Table 2, Table 3, Table 4, Table 5 and Table 6 below. The extracted fields comprised reference (first author and year), animal species and strain, model type (genetic, chemical/teratogenic, or surgical), induction method or surgical protocol with defect size and location, age at intervention and at evaluation, lethality and survival rate, induction rate, cleft type and laterality, and key methodologic insights relevant to model fidelity. Two investigators independently performed the data extraction; in cases of discrepancy, a third investigator was consulted to reach consensus. Extracted data were cross-checked against the original full-text articles before final tabulation.

## 3. Cleft Lip Models

A cleft lip is a unilateral or bilateral gap between the lateral side of the upper lip and philtrum, with most cleft lips followed by a cleft palate [27]. Isolated cleft lip has an incidence of 15% of all cleft types [64]. Although cleft lip significantly impacts patients globally as one of the most common congenital diseases [65], few animal models have been established to investigate the cleft lip: only two genetic animal models and one chemical-induced animal model have been established to investigate the relations between certain genes and their role in lip fusion and formation and cleft lip (Table 1).

The CRISPR/Cas9-mediated *Growth arrest and DNA damage 45G* (*GADD45G*) rabbit model is the only model that creates a cleft lip without alveolar cleft and/or cleft palate [27]. With an induction rate of 100%, the cleft lip occurs as either bilateral (90%) or unilateral (10%), and the consistency of cleft severity is relatively poor. Due to suckling problems, 100% of rabbits with a cleft lip in this model died within 3 days of birth.

For the other two animal models, one being genetic [*Twirler (Tw)* gene knockout in C57BL/6 mice] [28] and one being chemical-induced (2,3,7,8-tetrachlorodibenzo-p-dioxin (TCDD) orally administered to A/J mice) [29], both presented as cleft lip with alveolar cleft and cleft palate (CL + AC + CP) (Table 1). While the *Tw*-knockout has a 100% induction rate for CL + AC + CP, it also presents variations among embryos, with 69% of the embryos exhibiting bilateral cleft and 31% of the embryos exhibiting unilateral cleft [28]. In addition, CL + AC + CP is not the only feature of *Tw*-knockout mice. Due to severe craniofacial defects, such as ear and nasal cavity defects, all the pups died within 24 h after birth [28]. A chemical model was also utilized to enhance understanding of the mechanism by which TCDD induces cleft lip in three mouse strains: A/J, ICR, and C57BL mice [29]. The experiment with A/J mice found that cleft lip occurred at a 4% frequency when TCDD was administered on E12, and in the control group (without TCDD), and the induction frequency did not increase with increasing TCDD dosage [29]. In addition, the clefts successfully induced in A/J embryos were bilateral defects [29]. Interestingly, no cleft lip was induced by TCDD administration in the ICR and C57BL mouse groups [29].

Overall, the currently reported animal models of cleft lip are limited to prenatal evaluation due to high postnatal mortality rate, which limits postnatal observations on craniofacial growth and development and the ability to perform postnatal cleft lip repair to mimic the clinical scenario. In addition, a large variation in the severity of the cleft occurred among embryos of each type of animal model, making it difficult to compare treatment effects among different strategies.

## 4. Alveolar Cleft Models

An alveolar cleft is due to the abnormal primary palate formation during gestation [32]. There are ongoing controversies regarding the effectiveness in aiding successful alveolar cleft repair and the potential long-term effects of bone-substitute materials for bone grafting [32]. To investigate and evaluate the various types of graft therapies, alveolar cleft animal models are predominantly induced through surgical approaches in comparison to congenital clefts due to the ability to create standardized defects through surgery [32] (Table 2).

Common species selected for the surgical animal models include rats, rabbits, dogs, and rhesus monkeys. While the alveolar cleft induction rate is 100% with the surgical approach, the survival rate varies among different models (Table 2), with relatively high mortality in rats, possibly due to respiratory problems, circulatory issues, and poor homeostatic control.

In addition, it is worth noting the large variation in the location and size of the clefts across animal models, with small animal models more intended to be unilateral [30,31,33,34], and large animal models more intended to be bilateral [39,40,41,43,44] (Table 2). Interestingly, when the unilateral model was created, more study groups selected the left side of the animal [30,33,35,36,37,43], which may be because left-sided clefts are more prevalent in humans [66]. In terms of the defect sizes, while the large animal models show relative consistency within each species, size was not standardized across the small rodent models. More specifically, the defect sizes ranged from 3 × 2 × 1 mm^3^ [30] to 7 × 4 × 3 mm^3^ [34] in the reported rat models (Table 2).

**Figure 2:**
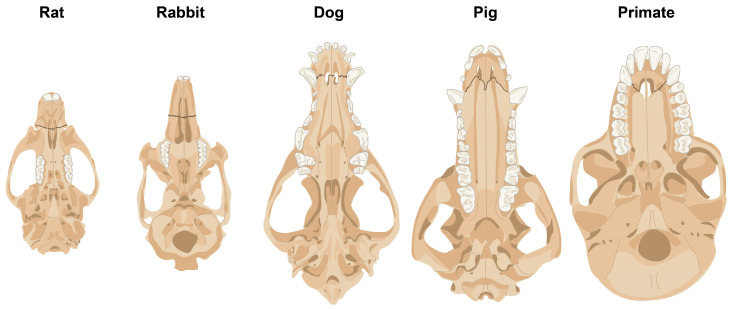
Demonstration of the location of the premaxillary–maxillary suture (black line) in each animal species used for alveolar cleft models.

Dogs, pigs, and monkeys have dental formulae that are relatively similar to humans, consisting of incisors, canines, and multiple premolars/molars. When surgically creating an alveolar cleft, the most common approach is to remove the second incisor and the alveolar wall around the extraction socket, and to extend vertically to the nasal cavity. The large defect size in large animal models also granted the surgical access to create the oronasal fistula by suturing the mucosa of the nasal and oral sides [38,39,40,42,43,44]. These surgical procedures allow the researchers to closely mimic the clinical condition of an alveolar cleft, which normally involves a congenitally missing maxillary lateral incisor at the cleft site. However, when considering the underlying mechanism of congenital alveolar cleft, the failure of the fusion of the pre-maxillary suture is the root, not the absence of the lateral incisor. Thus, we further looked into the location of the surgically created alveolar cleft in relation to the premaxillary–maxillary suture and revealed that while 100% of both pig [42,43] and monkey [44] models had involved disrupting the premaxillary–maxillary suture, only 50% of dog models [40,41] did so. This is due to the fact that the suture between the incisive bone/premaxilla and the maxilla is located adjacent to the maxillary canine, not to the second and/or third incisors, in pigs and dogs [43,67] (Figure 2). Unless extending the surgical defect distally, extracting the second and/or third incisors alone would not involve disrupting the premaxillary–maxillary suture.

The influence of the anatomic difference between human beings and the experimental models on the clinical relevance of the alveolar cleft models is further magnified in rabbits and rats, as both of them have a long span of the alveolar process between incisor and posterior teeth, and the premaxillary–maxillary suture is presented in the center of the alveolar process rather than adjacent to any teeth [68] (Figure 2). Thus, creating an alveolar cleft with the incisor or first molar as the anatomical reference landmark led to the low percentages of rabbit (33%) and rat (40%) alveolar cleft models that involved disrupting the pre-maxillary suture [33,34,35], which do not fully represent the naturally established human alveolar defects, possibly compromising comprehensive treatment and reconstructive therapy studies.

In addition to the variation occurring in the location and the size of the defect within each species, differences are also presented on whether to close the defect with a gingival flap, whether to fill the defect with bone wax or iodoform gauze, and whether to involve the nasal floor to create the oronasal fistula (Table 2). Collectively, these variations indicate persistent heterogeneity in surgical design and a lack of standardized protocols for creating experimental alveolar clefts, particularly regarding the use of an oronasal fistula. It is worth noting that an oronasal fistula is a critical postoperative complication in CL/P management [69]. Its clinical significance extends beyond mere anatomical failure; fistula creates a persistent communication between the oral and nasal cavities, leading to nasal regurgitation of fluids and food, hypernasal speech due to velopharyngeal insufficiency, and chronic nasal infections or discharge [69]. Moreover, recurrent rhinitis and otitis media with effusion may exacerbate hearing loss, further compromising speech and language development in pediatric CLP patients. The presence of a fistula also imposes psychological burdens, including social embarrassment and feeding difficulties that may lead to poor nutritional status [70]. From a surgical standpoint, oronasal fistulas complicate subsequent alveolar bone grafting, orthodontic treatment, and definitive nasal reconstruction, often requiring multiple revision procedures with unpredictable success rates [71,72]. Consequently, preventing, identifying, and managing oronasal fistulas is a cornerstone of contemporary CLP care, driving research into surgical technique refinement, tissue engineering, and adjunctive wound healing therapies. Thus, further studies on how to create animal models that can simulate and repair these specific defects are crucial.

Despite the large amount of available alveolar cleft models, they are composed mainly of adult animals; for instance, 7–16 week-old rats [30,31,32,33,34], young adult rabbits (specific age unclear) [35,36,37], 5–6 month-old Bama miniature pigs [43], 6–10 year-old Rhesus monkeys [44], and 22-month-old dogs were chosen for the surgeries [40], with exceptions of 85 and 95–100 day-old dogs [38,39,41], 5 week-old pigs [42], and 3–4 week-old rabbits [35] that were selected for five studies (Table 2). While adult animals may be excellent models for evaluating the biocapacity of different grafting materials, their use limits the ability to examine how surgical intervention and grafting techniques influence craniofacial growth and alveolar development during juvenile growth phases. In other words, current models provide limited insight into the developmental implications of cleft reconstruction strategies. Moreover, in the few models that used younger animals [38,39,41], their defects deviate from the premaxillary–maxillary suture, further limiting the applicability of these models to naturally occurring alveolar clefts in humans.

## 5. Midpalatal Cleft Models

Numerous experiments have been published on the replication of the midpalatal cleft. Due to the intensive number of articles, the reported midpalatal cleft models are summarized based on the induction techniques, namely the chemically induced model (Table 3), genetic mutant model (Table 4), and surgical model (Table 5), as listed below in each subsection.

### 5.1. Chemically Induced Midpalatal Cleft Models

Chemical teratogens have played a central role in elucidating the molecular and structural requirements for normal palatal development (Table 3). Among these, retinoic acid (RA) is the most widely used agent, consistently inducing midpalatal clefts across mouse and rat strains [45,46,47,48,49,50,51]. The teratogenicity of RA depends strongly on dose, timing, and isomer, with all-trans RA inducing >90% clefts at 100 mg/kg, whereas 13-cis RA requires a much higher dose of 400 mg/kg to achieve a comparable effect of 80% cleft induction [46]. In addition, the timing of RA administration is particularly critical. Abbott et al. showed that 100 mg/kg RA exposure at E9.5 induced 100% cleft formation, whereas only 28% cleft formation was induced if the exposure was postponed to E11.5, underscoring the heightened vulnerability of early palatal morphogenesis [49]. Later work demonstrated that RA not only suppressed the epithelial–mesenchymal signaling pathways such as the BMP family, which is essential for palate shelf growth and elevation [45,51], but also interrupted the rugae morphology and the timing of medial edge epithelial apoptosis [47,48], resulting in impaired palatal shelf adhesion and union [47].

Other teratogens highlight additional mechanistic pathways. Cortisone acetate induces near-universal cleft formation in A/J mice (99%) with much lower induction rates in B6 and B10 mice, emphasizing the strong interplay between teratogen exposure and genetic background [52]. TCDD (2,3,7,8-tetrachlorodibenzo-p-dioxin) similarly disrupts palatal fusion, inducing 90–100% cleft formation in A/J, C57BL/6J, and ICR strains, but its effective teratogenic dose lies extremely close to its lethal range—especially for A/J mice, which reach 100% fetal mortality at 40 μg/kg—making reproducibility difficult [29].

The 6-aminonicotinamide (6-AN) model in Sprague-Dawley rats demonstrates how mechanical obstruction can also cause cleft palate. Depending on fetal nutrition and dosage, 6-AN induces mandibular underdevelopment and tongue malposition due to the small mandible, resulting in delayed or failed palatal elevation from the physical blockage of the displaced tongue, with fasting worsening the cleft outcome [53]. This spectrum of partial to complete midline clefts parallels features of the Pierre Robin sequence, reinforcing the multifactorial nature of palatogenesis.

Finally, intramuscular dexamethasone administered between E13 and 16 induced a midpalatal cleft in New Zealand rabbits, and offers an unusual advantage among teratogenic models, postnatal survival, as newborn rabbits can be hand-reared, making this model valuable for postnatal surgical and regenerative studies [54]. However, the high lethality rate and low cleft induction rate indicate the need for further optimization of this model.

Together, chemically induced models reveal the wide-ranging vulnerabilities of palate development, ranging from molecular signaling disruptions to mechanical interference. Notably, the majority of the models had the cleft evaluations conducted on the prenatal embryonic stage, highlighting the inherent limitations of teratogen models for long-term translational work.

### 5.2. Genetic Mutant Models of Midpalatal Cleft

Genetic mouse models have been instrumental in defining the molecular architecture of palatogenesis (Table 4), demonstrating that cleft palate can result from disruptions in epithelial fusion, mesenchymal proliferation, craniofacial patterning, and endocrine signaling.

Several models center on deficiencies in mesenchymal proliferation and signaling. *Msx1*-knockout mice exhibit severe midpalatal clefts accompanied by pronounced craniofacial and dental abnormalities, such as absence of the alveolar process and maxillary incisors, as well as abnormalities in the middle ear, reflecting the gene’s essential role in maxillary arch development and tooth formation [59]. Similarly, *Titf2*-knockout mice present with a complete cleft palate coupled with thyroid dysgenesis, demonstrating how endocrine and craniofacial developmental pathways intersect [57].

The process of epithelial seam breakdown is highlighted in models lacking *TGF-β3*, where the medial edge epithelium (MEE) persists and prevents palatal fusion [60]. These mice show both complete and incomplete clefts with early perinatal lethality [60].

Other models affect palatogenesis by influencing craniofacial morphology. *Gli3*-knockout mice, for example, exhibit high cleft rates attributable to abnormal tongue posture and delayed palatal elevation, a mechanical barrier to fusion [55]. Meanwhile, epithelial overexpression of Sonic hedgehog (*SHH*) halts palatal fusion from failed palatal elevation and distorts tooth germ proliferation, recapitulating features of nevoid basal cell carcinoma syndrome and revealing the consequences of excessive epithelial–mesenchymal signaling [56].

Collectively, these genetic models show that, while many pathways can lead to cleft palate, disruptions in epithelial fusion and mesenchymal proliferation are especially potent. However, because many genetic mutants exhibit near-universal perinatal lethality, their usefulness is largely restricted to mechanistic embryologic studies, which are not extended to postnatal or regenerative research.

### 5.3. Surgically Created Midpalatal Defects

The early animal surgical models of midpalatal cleft were conducted on large animals, which provide additional insights regarding functional consequences (Table 5). For instance, in rhesus monkeys, surgically created midpalatal clefts produced persistent alterations in Eustachian tube function and high rates of otitis media with effusion, paralleling the clinical association between cleft palate and middle ear disease [62]. In beagle dogs, researchers demonstrated that extensive palatal excision and flap manipulation can impair maxillary growth and dentoalveolar development, an important surgical consideration for pediatric cleft care [61].

However, due to ethical considerations, small animal models are developed for evaluating regenerative materials. In skeletally mature rats, a standardized critical-sized (7 × 2.5 × 1 mm^3^) midpalatal defect was created on the premaxilla, with the consistent defect geometry and healing patterns confirmed by micro-CT imaging, leaving a persistent central void ideal for testing bone grafts and scaffolds [33]. However, this model may not fully reflect the clinical condition, as the rats have a long premaxilla that is far from the posterior dentition. Rabbit models further refine this approach by assessing how defect size, defect location, and surgical technique influence outcomes. Researchers compared anterior, half-palatal, versus complete hard-palate resections, finding that small and moderate defects showed substantial spontaneous regeneration, while complete removal yielded a stable, non-healing midpalatal cleft. Their use of piezoelectric instruments improved precision and minimized soft-tissue damage, enhancing reproducibility for biomaterial experimentation [35].

Although surgical models do not replicate the embryologic origins of cleft palate, they allow postnatal survival, controlled experimentation, and systematic evaluation of regenerative strategies, making them indispensable within the translational research pipeline. However, the currently available surgical models of midpalatal cleft are limited, especially compared with the variety of alveolar cleft model designs. Further studies are needed to identify the optimal model for surgically induced midpalatal cleft, especially in young animals, to allow the evaluation of the impact of cleft on postnatal growth of the craniofacial structure.

## 6. Cleft Soft Palate Models

A cleft soft palate is where the muscles in the soft palate are not fused properly, with the levator veli palatini and tensor veli palatini muscles (the velopharyngeal muscles) primarily impacted in cleft soft palates (in comparison to hard palate clefts) [63]. Failure of palatal fusion in the soft palate can have serious effects on individuals’ speech, respiration, and middle ear function, among others [63].

Cleft soft palate defects have a multifactorial etiology involving both environmental and genetic factors. To understand the underlying molecular and developmental mechanisms, currently, there are two genetic mouse models that have been established to focus on soft palate clefts (Table 6). When knocking out *TGF-β3*, defective palatogenesis and delayed pulmonary development were observed, resulting in 100% secondary clefts in mouse pups, with 56% of the pups presenting with a cleft soft palate [60]. The pertinent role of TGF-β in proper soft palate fusion is further validated with the basal epithelial-specific deletion of the TGF-β signaling mediator Smad4 (*Smad4^fl/fl^*; *K14-Cre*; *Irf6^+/R84C^*), which leads to 100% induction of submucous midpalatal cleft [63]. Lacking *Smad4* expression in MEE resulted in failed MEE apoptosis during palatal fusion, which can be rescued by elevated *Irf6* expression [63]. Neither of the existing genetic models conducted postnatal observations, especially with the *TGF-β3* knockdown resulting in perinatal death. A new study or animal model would be needed to further evaluate the long-term impact and recovery in relation to Smad4 and TGF-β3.

A condition that commonly accompanies cleft palates in infants is otitis media with effusion (OME), and this condition improved in affected infants after surgical repair of the cleft palate [62]. To further understand the relationship between OME and cleft soft palate, a surgical model of cleft soft palate had been established with four juvenile and adult rhesus monkeys (Table 6), with an incision on the palatal midline from the uvula to the posterior border of the palatine bone [62]. After surgery, 87.5% of the ears of monkeys with partial soft palate cleft had developed a recurrent OME, aligning with the universal finding of OME observations in infants with unrepaired cleft palate [62]. However, this study did not perform any other craniofacial evaluations to observe the potential impact of soft palate cleft on postnatal craniofacial growth and development.

## 7. Discussion

Existing animal models for CL/P conditions include congenital approaches (genetic or in utero teratogenic) and surgical approaches. Congenital models can be informative mechanistically but often require substantial technical expertise, can be associated with fetal loss and additional malformations, and frequently show wide variability in cleft phenotype [20,21]. Practical limitations also arise after birth: pups with obvious craniofacial defects may receive less maternal care, and in some contexts, maternal rejection or cannibalism has been reported, complicating postnatal follow-up [21,73]. Thus, the currently available articles limited their evaluations to the prenatal stage for developmental biology. In addition, the embryonic morphogenesis was always conducted with bright-field and scanning electron microscopy, while the molecular evaluation was always conducted with histology (Table 7).

The surgical approaches, on the other hand, can offer excellent control over defect dimensions and location, and are commonly used to test grafting materials. Specifically, the surgical survival rate, defect healing (assessed by micro-CT and histology), and the presence of other complications (such as otitis media) are the common evaluation methods. In the early era of cleft studies, large-animal surgical models in dogs and nonhuman primates have been used to study growth and repair, but their cost, housing needs, anesthesia complexity, and the challenges of cell-based studies—often requiring immunosuppression—reduce feasibility and complicate interpretation [74,75,76,77,78]. To respond to the needs in translational research of CL/P, rat and rabbit surgical models have expanded substantially over the past decades, but many published alveolar cleft/defect paradigms are created in skeletally mature animals and at sites that do not correspond to the true embryologic fusion interface between the premaxilla and maxilla, including models based on incisor or molar extraction or other convenient alveolar locations [32,34,36,79,80,81] (Table 7).

Incorporating multiple assessments of model validity into an animal model substantially elevates its rigor, moving beyond simple performance metrics to address whether a model measures what it claims to and serves its intended purpose [82,83]. These include: face validity, which evaluates if the animal model looks like the human condition on the surface, or if the animal exhibits the same anatomical defect as humans; construct validity, which evaluates if the animal model arises from the same underlying cause (genetic, molecular, or environmental) as the human condition, or does the animal model share the same pathogenesis; last but not least, predictive validity, which evaluates if the animal model correctly predicts what will happen in humans, as the response to a prevention strategy or the success of surgical/rehabilitative techniques. Application of these validity criteria is currently limited to animal models of human mental disorders [83,84,85]. However, adapting this concept to critically evaluate the craniofacial animal models would greatly advance the translational research in the craniofacial field.

In addition, when selecting an appropriate CL/P animal model, researchers should weigh not only scientific validity but also its impact on each of the 3Rs. For example, with the novel developments in the field of organ-on-a-chip, researchers may consider pursuing replacement using these novel in vitro models for genetic screenings before moving to mammalian models. For reduction, using inbred strains, prenatal screening to identify clefts early, and pilot power analyses minimize the number of animals needed. Refinement could be achieved by considering soft diets or orogastric feeding for pups with clefts, and by ensuring adequate analgesia. Finally, animal burden—the cumulative pain, distress, and lasting impairment—can be lowered by choosing unilateral over bilateral clefts, and surgical models over high-dose teratogen induction whenever the research question permits.

## 8. Conclusions

In summary, with the variety of reported animal models on CL/P, there are still various gaps from the currently available models to the real-life clinical scenarios, motivating future studies to generate novel models that create a defect at a developmentally relevant age and at a fusion-relevant anatomic site and thus evaluate postnatal growth effects and repair outcomes in a clinically meaningful way. For instance, the defect must be created with anatomical precision at the specific fusion sites—between the medial nasal and maxillary processes for cleft lip, between the premaxilla and maxilla for alveolar cleft, and between the left and right palatal shelves for midpalatal cleft. In addition, the extent and the size of the cleft should be controllable and consistent to allow comparisons among batches. After defect creation, a period of unperturbed postnatal growth must be allowed to evaluate natural craniofacial deformations (e.g., maxillary retrusion, arch collapse) using serial micro-CT and geometric morphometrics, followed by surgical repair at a clinically analogous age (about 6-month-old for cleft lip repair and about 9–12-year-old for alveolar cleft repair in humans). A worldwide collaboration is needed to achieve this task.

## Figures and Tables

**Table 1:** The list of animal models of cleft lip. CL: cleft lip; AC: alveolar cleft; CP: cleft palate; GADD45G: growth arrest and DNA damage 45G; TCDD: 2,3,7,8-tetrachlorodibenzo-p-dioxin; E: embryonic day.

Cleft Type	References	Species	Model Type	Model	Lethal	Induction Rate	Cleft Type
CL	Lu et al. (2019) [27]	Rabbit (New Zealand)	Genetic	CRISPR/Cas9-mediated *GADD45G* KO mediated on E13–E15	100% of rabbits die within 3 days after birth due to a suckling problem	100%	Bilateral (90%), unilateral (10%)
CL + AC + CP	Gong et al. (2000) [28]	Mice (C57BL/6J)	Genetic	*Twirler* KO	100% of pups die 24 h after birth	100%	Bilateral (68.75% [11/16]), unilateral (31.25% [5/16])
CL + AC + CP	Yamada et al. (2006) [29]	Mice (A/J)	Congenital/Chemical	TCDD (0–40 μg/kg, oral) administered on E11.5–E12.5	Fetuses’ mortality rate: 100% (40 μg/kg); 50% (20 μg/kg); 20% (0 μg/kg)	4% in either the TCDD administration or the control groups	Bilateral

**Table 2:** The list of animal models of alveolar cleft. All the models were created surgically.

References	Species	Model	Age	Lethal	Cleft Type	Defect Involves the Pre-Maxillary Suture?	Presence of Oronasal Fistula?
Mohlhenrich et al. (2021) [30]	Rat (Wistar), male	2.70 ± 0.46 mm × 2.01 ± 0.25 mm × 1.18 ± 0.20 mm, between the pre-maxillary suture and the first molar, filled with bone wax, incision closed.	8 weeks	14/33 died	Unilateral, left	no	no
Xu et al. (2010) [31]	Rat (Sprague-Dawley), male	4 mm × 4 mm × 3 mm, in the area of the maxillary first molar with first molar removal, incision closed.	7 weeks	90% survival rate from the surgery	Unilateral, right	no	no
Sun et al. (2015) [32]	Rat (Sprague-Dawley), male	4 mm × 4 mm × 3 mm, in the area of the maxillary first molar with first molar removal, incision closed.	8 weeks	All survived the surgery	Bilateral (right side untreated, left side filled with bone wax)	no	no
Mostafa et al. (2014) [33]	Rat (Sprague Dawley or Wistar)	5 mm × 2.5 mm × 1 mm, at the alveolar crest, incision closed.	8 weeks or 16 weeks	All survived the surgery	Unilateral, left	yes	no
Nguyen et al. (2009) [34]	Rat (Sprague-Dawley)	7 mm × 4 mm × 3 mm, incisor to zygomatic arch, incision closed.	8 weeks	0–10% intraoperative mortality	Unilateral	yes	no
Sun et al. (2019) [35]	Rabbit (New Zealand White), male and female	Model 1: left alveolar process, 1.00 ± 0.20 mm long × 0.50 ± 0.05 mm wide, filled with iodoform gauze. Model 2: removal of the left central incisor and the superior, lateral, and inferior walls of the extraction socket, filled with iodoform gauze.	3 to 4 weeks	All survived the surgery	Unilateral, left	Model 1—no Model 2—yes	no
Kamal et al. (2017) [36]	Rabbit (New Zealand White)	Removal of the left central incisor and the superior, lateral, and inferior walls of the extraction socket. Intact nasal mucosa. Wound filled with bone wax and left open.	8 weeks	All survived the surgery	Unilateral, left	no	no
El-Bokle et al. (1993) [37]	Rabbit (New Zealand White), male	1 cm wide, removal of the left central incisor and an accessory incisor. The cleft extends vertically from the floor of the nose to the level of the alveolar crest, mucosa of the nasal and oral sides sutured to the left with oronasal fistula.	young adult (2.5–3.0 kg)	2/22 died post-surgery	Unilateral, left	no	yes
Wu et al. (2010) [38]	Dog (breed unspecified)	10 mm × 15 mm, between the first and second incisors. Left with oronasal fistula.	8 weeks	All survived the surgery	Unilateral	no	yes
Ou et al. (2006) [39]	Dog (breed unspecified) male + female	15 mm wide between the primary lateral and the primary canine. Left with oronasal fistula.	12 weeks	All survived the surgery	Bilateral	no	yes
Pourebrahim et al. (2013) [40]	Dog (Mongrel)	Removal of 2 of the 3 incisors bilaterally and a 15 mm bone defect from crest to nasal floor. Left with oronasal fistula.	22 months	All survived the surgery	Bilateral	yes	yes
Yao et al. (2018) [41]	Dog (beagle) male	Removal of the primary third incisors bilaterally, bone removed from the distal of the primary second incisor to the mesial of the primary canine. Left nasal floor mucosa intact.	95–100 days	All survived the surgery	Bilateral	yes	no
Caballero et al. (2015) [42]	Pig (Yorkshire × Landrace × Large White bred to Hampshire × Duroc × Pietran)	Central incisor to canine, 1 cm, 1.7 cm, or 2 cm wide cleft, cleared to the nasal mucosa within the piriform aperture, empty or filled with different grafting materials, wound closed with suture.	5 weeks	All survived the surgery	Unilateral	yes	no
Zhou et al. (2022) [43]	Pig (Bama miniature pigs), male + female	Model 1: buccal to second incisor and canine, 1.8 cm in length, including the removal of the third incisor. Model 2: buccal to first and third incisors, 1.7–2 cm in length, including the removal of the second incisor. Wound closed with suture for both models	5–6 months	All survived the surgery	Unilateral left (model 1); Bilateral (model 2)	yes	no
El-Deeb et al. (1985) [44]	Primate (Rhesus Monkeys), female	1 cm wide, extraction of the maxillary lateral incisors and canines, and removal of the buccal and palatal bone. Oronasal fistula, with oronasal tube filled with acrylic resin.	6–10 years	All survived the surgery	Bilateral	yes	yes

**Table 3:** The list of chemically induced animal models of midpalatal cleft. RA: retinoic acid; TCDD: 2,3,7,8-tetrachlorodibenzo-p-dioxin; 6-AN: 6-aminonico-tinamide; DXM: Dexamethasone; IM: intramuscular injection; IP: intraperitoneal injection; NR: not reported; E: embryonic day; P: postnatal day.

References	Species	Model	Age of the CL/P Animal	Lethal	Induction Rate
Lu et al. (2000) [45]	Mice (BALB/c)	RA (100 mg/kg, oral) administered on E12.	evaluations conducted on E13-P1	NR	100%
Kochhar et al. (1984) [46]	Mice (ICR)	All-tans RA 25–200 mg/kg or 13-cis RA 100–400 mg/kg (oral) administered on E10.5, E11, or E11.5.	evaluations conducted on E17.5	Depending on the dosage and day of RA administered, the range is from 2 to 12%	>90% with 100 mg/kg dosage of all-trans RA on E11.5; 80% with 400 mg/kg for 13-cis administered on E11.5
Cuervo et al. (2002) [47]	Mice (CD-1)	RA (100 mg/kg, oral) administered on E14.2.	evaluations conducted on E18.5	NR	73% (14/19)
Horie & Yasuda (2001) [48]	Mice (Jcl:ICR)	RA (0.08–80 mg/kg, oral) administered on E10, E11, or E12.	evaluations conducted on E18	Depending on the dosage and day of RA administered, with the most effective dosage of 80 mg/kg administered on E11, there was 3.8% (3/79) dead fetuses	Depending on the dosage and day of RA administered, with the most effective dosage of 80 mg/kg administered on E11, there was an induction rate of 97.3%
Abbott et al. (1989) [49]	Mice (C57BL/6N)	RA (100 mg/kg, oral) administered on E9.5 or E11.5.	evaluations conducted on E13.5, E14.5, or E15.5	NR	RA exposure on E9.5 resulted in 100% induction (42/42 embryos); RA exposure on E11.5 resulted in 28% induction (36/127 embryos)
Ikemi et al. (2001) [50]	Rat (Crj:CD (SD))	RA (1.25, 5, 20, and 80 mg/kg, oral) administered on E13.5.	evaluations conducted on E19.5	0%	8% (6/75) for 20 mg/kg dosage of RA; 87% (69/79) for 80 mg/kg dosage of RA
Choi et al. (2011) [51]	Rat (Sprague-Dawley)	RA (80 mg/kg, IP) administered on E11.	evaluations conducted on E13–E17	NR	>90%
Bonner & Slavkin (1975) [52]	Mice (A/J, B6, B10, B10A)	Cortisone acetate (2.5 mg, IM) administered on E10.5–E13.5.	evaluations conducted on E16.5	NR	99% of A/J mice embryos; 25% for B6; 22% for B10; 81% for B10A
Yamada et al. (2006) [29]	Mice (A/J, C57BL/6J, ICR)	TCDD (0–40 μg/kg, oral) administered on E11.5–E12.5.	evaluations conducted on E18.5	Fetuses at 40 μg/kg: C57BL/6J and ICR mortality rate was less than 10%, A/J mortality rate was 100%; 20 μg/kg: A/J mortality rate was 50%; 0 μg/kg: A/J mortality rate was 20%	A/J: 90% of surviving fetuses at 20 μg/kg; C57BL/6J: 90% at 40 μg/kg; ICR: 100% at 40 μg/kg
Diewert (1979) [53]	Rat (Sprague-Dawley)	6-AN (IP) administered on E15. ANL group: regular diet + 8 mg/kg 6-AN; ANS group: fasted + 8 mg/kg 6-AN; ANH group: regular diet + 16 mg/kg 6-AN.	evaluations conducted on E16–E18	NR	On E18: ANL group: 100% plate fusion, with 80% had small rostral clefts and 30% had small rostral and caudal clefts; ANS group: 100% elevation, with 37% had full cleft and 63% had partial cleft; ANH group: 100% complete midline cleft, with 87% had full elevation and 13% had partial elevation
Liu et al. (2021) [54]	Rabbit (New Zealand)	DXM (1 mg, IM) administered on E13–E16.	evaluations conducted on P30	34.4% (25/73)	60.4% (29/48) in survivors

**Table 4:** The list of genetic mutant animal models of midpalatal cleft. *Gli3*: GLI family zinc finger 3; *Shh*: Sonic hedgehog signaling molecule; *Titf2*: also called *FoxE1* (forkhead box E1); *Ap2β1*: adaptor related protein complex 2 subunit beta 1; *Msx1*: msh homeobox 1; *TGF-β3*: transforming growth factor beta 3; KO: knockout; NR: not reported; E: embryonic day; P: postnatal day; h: hours.

References	Species	Model	Age of the CL/P Animal	Lethal	Induction Rate
Huang et al. (2008) [55]	Mice (C57/BL6)	*Gli3* KO	evaluations conducted on E13.5–E18.5	NR	generation backcrossed into C57/BL6 strain: 1st generation—7/22 (32%), 2nd generation—7/10 (70%), 3rd generation—16/17 (94%)
Cobourne et al. (2009) [56]	Mice (CBA/C56 BL6)	K14-Cre *Shh* overexpression	evaluations conducted on E13.5–E17.5	perinatal lethality	34/42 (81%)
Felice et al. (1998) [57]	Mice (C57BL/6)	*Titf2* KO	evaluations conducted on P0	died within 48 h after birth	100%
Li et al. (2010) [58]	Mice (FVB/NJ)	*Ap2β1* KO	evaluations conducted on E18	100% perinatal death	25%
Satokata et al. (1994) [59]	Mice (BALB/c or C57BL/6J)	*Msx1* KO	evaluations conducted on E11.5, E14.75, E17.75, P0	died within 24 h after birth	100%
Koo et al. (2001) [60]	Mice (Swiss Webster)	*TGF-β3* KO	evaluations conducted on E18.5	lethal in the early perinatal stage	4/9 (44%)

**Table 5:** The list of surgically created animal models of midpalatal cleft. NR: not reported; E: embryonic day; P: postnatal day; h: hours; g: gram; kg: kilogram.

References	Species	Model	Age of the CL/P Animal	Lethal
Mostafa et al. (2014) [33]	Rat (Wistar)	7 × 2.5 × 1 mm^3^ defect in the premaxilla, mucosal flaps were closed.	16 weeks old (375–400 g)	All survived surgery, euthanized 8 weeks post-op
Sun et al. (2019) [35]	Rabbit (New Zealand White)	Model 1: removal of the anterior half of the hard palate; Model 2: removal of the left half of the hard palate; Model 3: removal of the entire hard palate.	3 to 4 weeks old	All survived surgery, euthanized 8 weeks post-op
Meijer et al. (1978) [61]	dog (beagle)	Excised the central strip of mucoperiosteum and a similar 5 mm wide strip of bone. From 5 mm distal to the incisive foramen to the level of the hamulus, including the whole palate. Left the distal soft palate intact.	7 weeks old	All survived surgery, euthanized 1-month post-op
Doyle et al. (1980) [62]	Rhesus monkey (Macaca mulatta)	5 mm cleft extending along the midpalatal suture from the posterior border of the palatine bone to the posterior border of the incisive foramen.	juvenile and adult (2.5–6.0 kg)	All survived surgery, 5–13 months follow-up

**Table 6:** The list of animal models of soft palate cleft. Smad4: SMAD family member 4; Irf6: interferon regulatory factor 6; TGF-β3: transforming growth factor beta 3; KO: knockout; NR: not reported; E: embryonic day; P: postnatal day; kg: kilogram.

References	Species	Model Type	Model	Age of the CL/P Animal	Lethal	Induction Rate
Iwata et al. (2013) [63]	Mice	Genetic	*Smad4^fl/fl^*; *K14-Cre*; *Irf6^+/R^*^84^	evaluations conducted on P0	NR	100%
Koo et al. (2001) [60]	Mice (Swiss Webster)	Genetic	*TGF-β3* KO	evaluations conducted on E18.5	Lethal in the early perinatal stage	5/9
Doyle et al. (1980) [62]	Rhesus monkey (Macaca mulatta)	Surgical	incision on the palatal midline from the uvula to the posterior border of the palatine bone	juvenile and adult, 2.5–6.0 kg	All survived from surgery, 5–13 months follow-up	100%

**Table 7:** The strengths and limitations of each category of cleft models.

Model Type	Strengths	Limitations	Common Methods for Outcome Evaluation	Intended Use
Chemically induced	Closely mimic the embryonic development of CL/P Easy to perform Do not need a target gene	The size and extent of the cleft cannot be controlled Fetal loss The induction rate varies Difficult for postnatal follow-up Always combined with other craniofacial deformities	Skeleton staining Bright-field images Scanning electron microscopy images Histology	Teratogenicity Developmental biology Craniofacial growth (prenatal)
Genetically engineered	Closely mimic the embryonic development of CL/P Informative mechanistically, especially for certain targeted genes	Require substantial technical expertise Need to have one or more target genes The size and extent of the cleft cannot be controlled Fetal loss The induction rate varies Difficult for postnatal follow-up Always combined with other craniofacial deformities Predominantly in mice	Skeleton staining Bright-field images Scanning electron microscopy images Histology	Developmental biology Craniofacial growth (prenatal)
Surgically created	Excellent control over defect dimensions and location 100% induction rate Low lethal rate Can be used to test different grafting materials	Currently available models have challenges in cell-based studies The majority created on skeletally matured animals, does not allow the evaluation of puberty growth and development Some models have a surgical site that does not correspond to the true embryologic fusion interface between the premaxilla and maxilla	Survival rate Micro-CT Histology Functional outcomes	Surgical repair Biomaterial testing Wound healing/scarring

## Data Availability

No new data were created or analyzed in this study. Data sharing does not apply to this article.
